# Locking plate osteosynthesis in displaced 4-part fractures of the proximal humerus: A systematic review of benefits and harms

**DOI:** 10.3109/17453674.2011.588856

**Published:** 2011-09-02

**Authors:** Stig Brorson, Lars H Frich, Annika Winther, Asbjørn Hróbjartsson

**Affiliations:** ^1^Department of Orthopaedic Surgery, Herlev University Hospital, Herlev; ^2^Department of Orthopaedic Surgery, Odense University Hospital, Odense; ^3^The Nordic Cochrane Centre, Rigshospitalet, Copenhagen, Denmark; Correspondence: sbrorson@hotmail.com

## Abstract

**Background and purpose:**

There is considerable uncertainty about the optimal treatment of displaced 4-part fractures of the proximal humerus. Within the last decade, locking plate technology has been considered a breakthrough in the treatment of these complex injuries.

**Methods:**

We systematically identified and reviewed clinical studies on the benefits and harms after osteosynthesis with locking plates in displaced 4-part fractures.

**Results:**

We included 14 studies with 374 four-part fractures. There were 10 case series, 3 retrospective observational comparative studies, 1 prospective observational comparative study, and no randomized trials. Small studies with a high risk of bias precluded reliable estimates of functional outcome. High rates of complications (16–64%) and reoperations (11–27%) were reported.

**Interpretation:**

The empirical foundation for the value of locking plates in displaced 4-part fractures of the proximal humerus is weak. We emphasize the need for well-conducted randomized trials and observational studies.

There is considerable uncertainty about the optimal treatment of displaced 4-part fractures of the proximal humerus (Misra et al. 2001, Handoll et al. 2003, Bhandari et al. 2004, Lanting et al. 2008). Only 2 small inconclusive randomized trials have been published (Stableforth 1984, Hoellen et al. 1997). A large number of interventions are used routinely, ranging from a non-operative approach to open reduction and internal fixation (ORIF), and primary hemiarthroplasty (HA).

In the last decade, locking plate technology has been developed and has been heralded as a breakthrough in the treatment of fractures in osteoporotic bone (Gautier and Sommer 2003, Sommer et al. 2003, Haidukewych 2004, Miranda 2007). Locking plate technique is based on the elimination of friction between the plate and cortex, and relies on stability between the subchondral bone and screws. Multiple multidirectional convergent and divergent locking screws enhance the angular stability of the osteosynthesis, possibly resulting in better postoperative function with reduced pain. Reported complications include screw cut-out, varus fracture collapse, tuberosity re-displacement, humeral head necrosis, plate impingement, and plate or screw breakage (Hall et al. 2006, Tolat et al. 2006, van Rooyen et al. 2006, Agudelo et al. 2007, Gardner et al. 2007, Khunda et al. 2007, Ring 2007, Smith et al. 2007, Voigt et al. 2007, Egol et al. 2008, Kirchhoff et al. 2008, Owsley and Gorczyca 2008, Brunner et al. 2009, Micic et al. 2009, Sudkamp et al. 2009). The balance between the benefit and harms of the intervention seems delicate.

Several authors of narrative reviews and clinical series have strongly recommended fixation of displaced 4-part fractures of the humerus with locking plates (Bjorkenheim et al. 2004, Hente et al. 2004, Hessler et al. 2006, Koukakis et al. 2006, Kilic et al. 2008, Korkmaz et al. 2008, Shahid et al. 2008, Papadopoulos et al. 2009, Ricchetti et al. 2009) and producers of implants unsurprisingly strongly advocate them (aap Implantate 2010, Stryker 2010, Synthes 2010, Zimmer 2010). Despite the increasing use of locking plates (Illert et al. 2008, Ricchetti et al. 2009), we have been unable to identify systematic reviews on the benefits and harms of this new technology in displaced 4-part fractures. Thus, we systematically identified and reviewed clinical studies on the benefits and harms after osteosynthesis with locking plates in displaced 4-part fractures of the proximal humerus.

## Methods

### Eligibility criteria

We included clinical studies, randomized trials, comparative studies (prospective or retrospective), and case series, involving patients with displaced 4-part fractures according to Neer (1970), or with valgus impacted 4-part fractures according to Jakob (1991). Eligible studies were those that had been designed to study outcomes in 4-part fractures after primary osteosynthesis with any type of locking plate within 2 weeks of injury, with a follow-up period of 6 months or more; evaluation of patients had to be done using the Constant-Murley shoulder score (1987).

We excluded studies of other fracture patterns (non-displaced, 2-part, 3-part, articular surface and head splitting; compound and pathological) and studies in children (age < 18). Furthermore, case series were excluded if the number of fractures included was less than 10.

### Search strategy

We performed iterative searches of PubMed, Embase, Web of Science, and the Cochrane Library, restricting it to the years 1999–2009 (week 42). We also searched the OTA Annual Meeting Search Engine (2010) and read abstracts and posters from annual OTA meetings held during the period 1999–2009. One of the authors read reference lists from all the studies that might be eligible. We did not contact authors, but we contacted implant providers for additional information on studies and data.

### Data extraction

One author scanned titles and abstracts for possibly eligible studies. 2 reviewers read the full-text version of potentially eligible studies, and decided independently on inclusion. Disagreements were solved by discussion. 2 authors independently extracted data on study characteristics and results using pre-tested forms. Disagreements were solved by discussion.

For any randomized trials identified, we had planned to assess the risk of bias using the Cochrane Collaboration's tool for assessing risk of bias. For observational studies, we assumed that risk of bias was lower in the studies that fulfilled the following criteria: (1) the cohort was consecutively or randomly sampled; (2) dropouts or loss to follow-up were few (< 15%); (3) we considered the classification procedure to be adequate; (4) outcome was assessed blind; (5) there were no conflicts of interest; (6) we considered the cohort to be fairly representative of a typical patient with a 4-part fracture. Case series were not assessed according to methodological quality.

### Operational definitions

We defined a “prospective, observational, comparative study” to be any study that collected data prospectively and compared outcomes in patients treated with plates and patients treated with a control intervention. A “retrospective, observational, comparative study” was similarly defined as a study in which outcomes were collected retrospectively, for example based on a clinical database. Furthermore, we defined a case series to be study of the outcome in a series of patients treated with locking plate, but with no comparison with patients who had received a control intervention.

## Results

The search yielded 1,008 references (Figure). The majority of the studies were clearly irrelevant on closer scrutiny. 4 studies reported data on 3- and 4-part fractures combined (Aprato et al. 2005, Hirschmann et al. 2007, Moonot et al. 2007, Illert et al. 2008), 9 studies classified according to the AO system exclusively (Plecko and Kraus 2005, Biasbetti et al. 2006, Agudelo et al. 2007, Babst and Brunner 2007, Roderer et al. 2007, Korkmaz et al. 2008, Sharafeldin et al. 2008, Erhardt et al. 2009, Sudkamp et al. 2009), and in 1 study the patients were not evaluated with the Constant-Murley score (Shahid et al. 2008). We included 14 clinical studies with 374 patients. There were no randomized trials, but we included 1 prospective observational comparative study, 3 retrospective observational comparative studies, and 10 case series.

**Figure:**
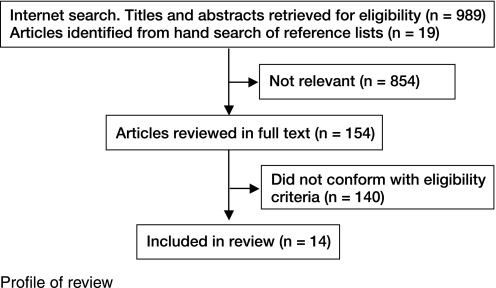
Profile of review

The risk of bias in the observational, comparative studies was considerable (Table). No studies were considered to have a low risk of bias in all 6 dimensions. Unclear reporting in all 14 studies hampered the assessment. In the prospective study, 1 dimension was scored as having a low risk of bias, and in each of the 3 other retrospective studies 3 dimensions were scored as having a low risk of bias.

**Table:** Risk of bias in the observational comparative studies

A	B	C	D	E	F	G
Krivohlavek (2008)	ns	no **^a^**	yes	no	ns	ns
Solberg (2009b)	yes	yes	no	no	ns	yes
Gradl (2009)	ns	yes	yes	yes **^b^**	ns	ns
Handschin (2007)	yes	yes	ns	no	ns	yes

**^a^** Young population, mean age 57.4 years (women 64.5 years; men 45.3 years).

**^b^** Classification by senior author.

ns: not specified. A Study B Cohort consecutively or randomly sampled C Cohort representative D Dropouts or loss to follow-up of < 15% E Classification procedure adequate F Outcome blindly assessed G No conflicts of interests

### Observational comparative studies—prospective

In Krivohlavek et al. (2008), 2 prospective cohorts of 19 locking plates (Philos) and 21 locking nails (Targon) were compared after 12 months. Mean Constant score compared to the contralateral side was 73 (SD not reported, range 18–100) for locking plates and 79 (SD not reported, range 43–100) for locking nails. The overall complication rate was 12/49 for locking plates (4 avascular necrosis, 2 infections, 4 impingement, and 2 periarticular ossification, not specified according to Neer category) as compared to 11/48 for locking nails.

### Observational comparative studies—retrospective

Solberg et al. (2009b) retrospectively compared outcome after 15 locking plate osteosyntheses (Synthes, Zimmer, Stryker) to 23 primary hemiarthroplasties (DePuy, Stryker, Zimmer). Non-adjusted mean Constant score after 3 years was 65 (SD 11) for locking plates and 60 (SD 6) for hemiarthroplasty. Overall complication rate was 19/38 patients (not specified according to Neer-category) for locking plates (6 avascular necrosis, 6 screw penetrations, 4 loss of fixation, and 3 infections) and 10/48 for hemiarthroplasty (not specified according to Neer category). 9/38 patients with locking plates were reoperated. Bias due to 13 dropouts of 51 could not be excluded, especially 8 patients with an incomplete follow-up. The authors also mentioned a risk of selection bias caused by the treatment algorithm, as the most complicated fractures were allocated to the hemiarthroplasty group.

Gradl et al. (2009) prospectively evaluated 2 cohorts of 16 locking plates (LPHP; Mathys) and 16 nails (Targon) including a retrospective matched-pair analysis after 12–14 months. Mean Constant score compared to the contralateral side was 76 (SD 19) for locking plates and 71 (SD 25) for locking nails. Complication rates were 22/76 in the locking plate group and 17/76 in the nail group. 9 patients with locking plates were reoperated.

Handschin et al. (2008) retrospectively compared outcome after locking plate osteosynthesis (Philos) (n = 10) to outcome after osteosynthesis with one-third tubular plates (n = 17). Non-adjusted mean Constant score after at least 17 months was 57 (SD 8) for locking plates and 57 (SD 12) for one-third tubular plates. Complication rates in 4-part fractures were 2/10 for locking plates (1 avascular necrosis and 1 impingement) and 4/17 for tubular plates.

### Case series

Helwig et al. (2009) reported outcome in 11 4-part fractures (including fracture dislocations) treated with locking plates (Philos or T-LCP). Non-adjusted mean Constant score was 76 for 4-part fractures and 78 for 4-part fracture dislocations (SD not reported). 7 complications occurred (2 screw penetration and 5 avascular necrosis).

Kettler et al. (2006) reported outcome in a prospective study involving 26 4-part fractures treated with locking plates (Philos). Age- and sex-adjusted mean Constant score was 66 (SD not reported for 4-part fractures). Complications occurred in 8 of the 26. 8 patients were reoperated.

Hente et al. (2004) reported outcome in a prospective study involving 11 4-part fractures treated with locking plates (Philos). Non-adjusted mean Constant score was 70 (SD 21). Age- and sex-adjusted Constant score was 75 (SD 19). Complications occurred in 7 patients (5 avascular necrosis and 2 periarticular ossification).

Björkenheim et al. (2004) reported outcome in a retrospective study involving 12 4-part fractures treated with locking plates (Philos). Non-adjusted mean Constant score was 60 (SD not reported, range 30–72). 3 cases of avascular necrosis were reported.

Frangen et al. (2007b) reported outcome in 16 4-part fractures treated with locking plates (Königsee). Non-adjusted mean Constant score was 71 (SD 11). Mean Constant score relative to the contralateral side was 79 (SD 18). 3 cases of avascular necrosis were reported.

Frangen et al. (2007a) reported outcome in a retrospective study involving 44 4-part fractures treated with locking plates (Königsee). Non-adjusted mean Constant score was 82 (SD 21) for anatomically correct repositioning. 14 cases of avascular necrosis were found (3- and 4- part reported together, n = 54). Accurate anatomical reduction was found to be more important than implant type (p < 0.05). Locking plating did not lead to significant improvement in functional outcome compared to other ORIF techniques (but statistical testing was not reported).

Klitscher et al. (2008) reported outcome in a retrospective study involving 12 4-part fractures treated with locking plates (Philos). Median Constant score was 69. 10 complications occurred; 3- and 4-part fractures were reported together (n = 30).

Martinez et al. (2009) reported outcome in a retrospective study involving 25 4-part fractures treated with locking plates (Philos). Non-adjusted mean Constant score was “poor” (1–55 p.) in 1; “moderate” (56–70 p.) in 3; “good” (71–85 p.) in 15, and “excellent” (86–100 p.) in 6. Four complications occurred (1 axillary nerve palsy, 1 malunion, and 2 cases of impingement).

2 other case series were designed to assess the effect of angulation (Solberg et al. 2009a) or type of surgical approach (Hepp et al. 2008), and not the effect of locking plate as such.

Solberg et al. (2009a) retrospectively compared outcome after locking plate osteosynthesis (Synthes, Zimmer, Stryker) according to initial angulation of the humeral head in varus (n = 10) or valgus (n = 19). Non-adjusted Constant score after at least 18 months was 61 (SD 9) for initial varus angulation as compared to 69 (SD 10) for initial valgus angulation. All fractures malreduced more than 20 degrees in varus postoperatively failed. Complication rates were 19/24 for varus displacement and 9/46 for valgus displacement, with 3- and 4-part fractures reported together (p < 0.01). The complication rate in preoperative varus displacement of more than 60 degrees was higher regardless of Neer category. A statistically significant correlation between humeral head angulation and Constant score was reported.

Hepp et al. (2008) compared outcome after locking plate osteosynthesis (LPHP, Mathys) using a deltopectoral approach at 1 center (n = 7) to that after an extended anterolateral deltoid-split surgical approach at another center (n = 5). Non-adjusted mean Constant score after at least 12 months was 64 for deltopectoral approach and 63 for deltoid-split approach. The overall complication rate was 20/83 (for 2-, 3-, and 4-part fractures together). The overall reoperation rate was 9/83.

### Contact with manufacturers of implants

9 companies that provided locking plates for fractures of the proximal humerus were contacted by e-mail in order to identify additional studies or to obtain unpublished data. 3 replies were received, but no additional studies or data were provided.

## Discussion

We found that the clinical studies assessing the effect of locking plate osteosynthesis in displaced 4-part fractures were few and small, with a considerable risk of bias. No randomized trials were identified. We did note, however, that complications and reoperations after locking plate synthesis were frequent.

The strength of our review is its comprehensive scope, including all major types of clinical investigations, and its thorough search strategy. The weakness is that the empirical clinical studies on this topic were scarce and not reliable. In addition, our assessment of the risk of bias is based on common sense—and not on empirical investigations—so it should be regarded as tentative. Furthermore, we excluded studies with pooled outcome data from 3- and 4-part fractures. This does not appear to have had a major influence on our results, however. Brunner et al. 2009) conducted a prospective, multicenter study of 158 implanted locking plates for all types of proximal humeral fractures. The overall complication rate was 35%, the most common cause being primary screw penetration.

We are unaware of any previously published systematic review of a similar nature. A systematic review from 2009 analyzed the benefits and harms of locking plates in 2-, 3-, and 4-part fractures (Thanasas et al. 2009). The authors identified only case series, and reported satisfactory outcome in 2- and 3-part fractures (mean Constant score: 77 and 76). The overall mean Constant score was 68. Avascular necrosis occurred in 15% of the 4-part fractures. The authors concluded that in selected cases there is a potential for locking plates to reduce the need for prosthetic replacement in osteoporotic, comminuted fractures.

We did not include studies using the AO classification system exclusively. Südkamp et al. (2009) prospectively followed 187 patients with proximal humeral fractures classified according to AO (and not according to Neer) and treated with locking plates. They reported overall complication rates of 34% and reoperation rates of 19% after 1 year follow-up. The authors could not generally recommend the use of locking plates.

The observational studies and the case series included in the present review showed that the use of locking screws in rigid plates has led to a new entity of complications in the treatment of proximal humeral fractures. The major harm was primary and secondary screw penetration. Primary (intraoperative) screw penetration of the humeral head is usually caused by poor surgical technique, and makes the construct prone to failure (Sudkamp et al. 2009). Secondary screw penetration or screw cut-out is related to the rigidity of the locked osteosynthesis. Cut-out can appear in severely comminuted fractures with poor bone quality (Brunner et al. 2009), a condition for which locking screws are also recommended.

The severity of the injury is related to the risk of avascular necrosis, which is considered a major complication after fracture of the proximal humerus, often resulting in painful dysfunction of the shoulder. Traditional plating has been associated with a high rate of avascular necrosis, and recent publications have also reported high rates of avascular necrosis after locking plate osteosynthesis (Solberg et al. 2009a). Interestingly, favorable functional results were reported in some series despite avascular necrosis (Bjorkenheim et al. 2004, Hente et al. 2004, Helwig et al. 2009). Avascular necrosis after plating does not appear to be directly related to the Neer category of fracture, but rather reflects the complexity of the fracture. If the head is deprived of its blood supply at the moment of fracture, restoration by any technique seems meaningless. In cases where the blood supply is compromised, open reduction and internal fixation with plates and screws may restore the anatomy of the proximal humerus but may further compromise the vascularity of the head, leading to avascular necrosis and poor outcome.

In a recent review (Thanasas et al. 2009), an avascular necrosis rate of 15% was considered lower than usually expected after treatment with locking plates. However, this rate was based on short-term follow-up. Most studies are limited to a 1-year follow-up, and the clinical effect of avascular necrosis may present later (Greiner et al. 2009). Gerber et al. (2004) reported partial or total humeral head necrosis in 12 of 34 cases after different internal fixation methods of 3-part and 4-part fractures, after a follow-up period of 5 years. Longer follow-up is therefore necessary to determine the rate of avascular humeral head necrosis. Varus instability is an immediate sign of malfunctioning of the osteosynthesis. It may arise early, due to inappropriate reduction or failure at the level of the medial extension of the humeral head. Retarded varus instability is inevitably associated with screw cut-out. This phenomenon is a major problem directly related to the use of locking plates.

Primary hemiarthroplasty has been recommended for the treatment of displaced 4-part fractures of the proximal humerus for more than 3 decades (Kontakis et al. 2008). A satisfactory functional outcome is rarely achieved, however, and complications such as nonunion and malunion of the tuberosities are common (Fialka et al. 2008).

The natural course of displaced 4-part fractures is unknown. Only a few, small series have been published, with conflicting conclusions (Leyshon 1984, Zyto et al. 1997, Ilchmann et al. 1998, Zyto 1998, Lill et al. 2001, Urgelli et al. 2005). However, 3 randomized multicenter trials including a non-surgical treatment arm are currently being conducted, and results are expected in the near future (Brorson et al. 2009, Handoll et al. 2009, Den et al. 2010).

In summary, there have been few clinical studies evaluating locking plate osteosynthesis in displaced 4-part fractures. Those published were small and had a high risk of bias. This precluded reliable estimates of benefits and harms, but we did note high complication rates and reoperation rates. The balance between benefits and harms is difficult to establish without further studies being done with low risk of bias. Thus, we cannot recommend the routine use of locking plate osteosynthesis for displaced 4-part fractures of the proximal humerus.
